# Modeling spatial acuity improves trap capture of western flower thrips, *Frankliniella occidentalis* (Thysanoptera: Thripidae)

**DOI:** 10.1093/jisesa/ieaf049

**Published:** 2025-05-13

**Authors:** Natalie S Roberts, Madelyn Jones, Farooq Shah, Tariq M Butt, William L Allen

**Affiliations:** Department of Biosciences, Faculty of Science and Engineering, Swansea University, Swansea, UK; Department of Biology, Lund University, Lund, Sweden; Department of Biosciences, Faculty of Science and Engineering, Swansea University, Swansea, UK; Department of Biosciences, Faculty of Science and Engineering, Swansea University, Swansea, UK; Razbio Limited, Bridgend, UK; Department of Biosciences, Faculty of Science and Engineering, Swansea University, Swansea, UK; Department of Biosciences, Faculty of Science and Engineering, Swansea University, Swansea, UK

**Keywords:** integrated pest management, acuity modeling, crop pests, visual resolution

## Abstract

Colored sticky traps are used for management of many common agricultural insect pests. Several recent studies have shown that traps can be improved by systematically considering properties of color vision for the target species. In the current study, we extend this approach to spatial vision, using information about the interommatidial angle of an agriculturally important insect pest, western flower thrips *Frankliniella occidentalis* (Pergande), to predict spatial resolution capabilities for a yellow flower pattern across a range of viewing distances. We tested the hypothesis that pattern sizes matching the spatial resolution capabilities of western flower thrips at a given viewing distance would outperform traps with mismatched pattern sizes by measuring the number of western flower thrips caught on sticky traps containing differently sized flower patterns resolvable at 5, 10, or 20 cm. We found an interaction between pattern size and viewing distance, with significantly more western flower thrips caught on traps when the predicted resolvable distance of the pattern matched the distance traps were placed from a central release point. We further tested the range over which trap patterns are effective in more complex viewing environments using commercial polytunnels. In polytunnel trials, we found that increasing the resolvable distance of patterns increased western flower thrips capture up to approximately 26 cm, after which western flower thrips capture decreased up to the maximal visible range tested (50 cm) in the absence of additional sensory cues. Together, these results show the utility of considering spatial vision in improving trap performance and offers functional insights to improve pest management in visual trap design.

## Graphical Abstract

**Graphical Abstract:**
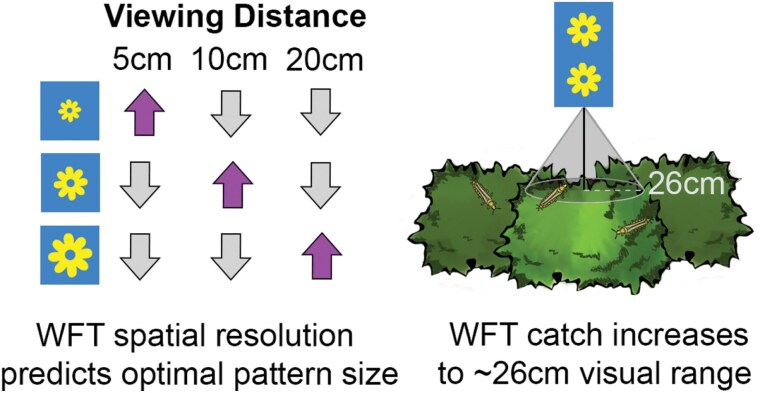

## Introduction

Vision is an important sensory modality in insect behavior, playing roles in many behaviors including host plant detection, feeding behaviors, and navigation (Reeves 2011, Kelber and Somanathan 2019, Van Der Kooi et al. 2021, Freas and Spetch 2023). Behavioral responses to visual stimuli are inherently shaped by underlying properties of the insect visual system. For example, chromatic vision depends on the number and types of photoreceptor classes in the eye (Terakita 2005), while factors such as the spacing and diameter of visual detectors impacts visual acuity (Dafni et al. 1997, Land 1997, Caves et al. 2018). Visual modeling uses these known features of a receiver’s visual system, along with general principles of vision, to predict visual function, which can ultimately provide a better understanding of insect behavior (Santer and Allen 2023, 2025).

Recently, visual modeling has been implemented in the design of color stimuli for pest management strategies. Colored sticky traps are an important part of integrated pest management strategies (Shimoda and Honda 2013, Mouden et al. 2017, Rodríguez and Coy-Barrera 2023) and many studies have assessed the role of trap color on insect catch, often finding a species-specific effect of color in trap success (Leong and Thorp 1999, Hoddle et al. 2002, Do Bae et al. 2015, Pobozniak et al. 2020, Csanády et al. 2021). However, while most studies rely on human perception of color for trap design, there has been a recent push to consider vision from the perspective of target species using visual modeling approaches (Santer and Allen 2023, 2025). Several empirical studies have demonstrated the effectiveness of visual modeling to improve trap design in a variety of insect species by selecting colors that are tailored to the visual system of the target species (Santer 2014, Dearden et al. 2023).

In addition to color, pattern elements can also impact the attractiveness of visual stimuli. The addition of pattern to sticky traps has been shown to improve trap capture for 2 economically important species of crop pest, western flower thrips (WFT) (*Frankliniella occidentalis*) and greenhouse whitefly (*Trialeurodes vaporariorum*) (Sampson et al. 2018, Dearden et al. 2023). However, the impact of trap pattern on insect attraction has been relatively understudied, particularly in the context of visual resolution capacity. An exception may be in pollinating insects, particularly bees, for which several studies have addressed the impact of spatial acuity on flower detection and pattern attraction (Hempel de Ibarra et al. 2015, Kelber and Somanathan 2019). Other examples include detection of conspecific color patterns in *Anolis* lizards (Fleishman et al. 2017) and cleaner shrimps (Caves et al. 2016), and detection of zebra stripe patterns in tsetse flies (Britten et al. 2016). Given the large variation in spatial acuity between species (ranging at least 4 orders of magnitude across animals with image-forming eyes), a consideration of the visual system in trap pattern design is critical to design patterns that are visible to target species (Caves et al. 2018, Feller et al. 2021). For example, trap patterns that are easily detectable to a human viewer, may be unresolvable for target species, particularly those with small eyes associated with poorer spatial resolution (Caves et al. 2018).

Detection of stimuli or pattern elements depends on the spatial acuity of the receiver’s visual system. For insect compound eyes, spatial acuity is determined in part by the angle between ommatidia (interommatidial angle), which depends on the number and size of ommatidia in the compound eye (Land 1997, Caves et al. 2018). Viewing distance also impacts whether a pattern is detectable, such that pattern details become less resolvable with increased distance, with smaller interommatidial angles increasing the distance that a stimulus can be resolved over (Land 1997, Caves et al. 2018). The minimum resolvable distance for a stimulus can, therefore, be calculated using the equation:


$$\begin{array}{l} D=\left(R/2\right)/\tan(\alpha /2) \end{array}$$ (1)

where *D* is the resolving distance, *R* is the stimulus size, and *α* is the interommatidial (IO) angle (Ben-Yakir et al. 2023).

In addition to anatomical properties of the eye, visual acuity can also be impacted by factors such as the lighting environment and movement, meaning that anatomical estimates of spatial acuity represent a theoretical upper limit of resolution power (Land 1997). Nevertheless, behavioral measures of spatial acuity closely match anatomical predictions in several cases (Northmore and Dvorak 1979, Miller et al. 1993, Northmore et al. 2007, Pettigrew and Manger 2008, Fleishman et al. 2017).

Traps with more than 1 color catch more WFT than those of a solid color (Dearden et al. 2023). This suggests that consideration of the spatial resolution capabilities of WFT could inform choice of pattern size in trap design. WFT are predicted to have low visual acuity, with only 60 to 70 ommatidia in each compound eye, resulting in interommatidial angle estimates between 10° and 14° (Lopez-Reyes et al. 2022, Ben-Yakir et al. 2023). In contrast, the interommatidial angles of many flying insects (eg Lepidoptera, Hymenoptera, Diptera) are between 1° and 3° (Land 1997), with the median interommatidial angles of a large-scale comparison of arthropods being 2° in the water-flea (*Polyphemus pediculus*) (Feller et al. 2021). This means that pattern sizes used in studies with many other species may be below the resolution threshold for WFT, making consideration of the WFT visual system critical in trap design.

In the current study, we design contrasting pattern sticky traps based on the predicted spatial resolution capabilities of WFT. Yellow flower patterns added to blue sticky traps were designed to be resolvable at 3 specific viewing distances and we tested whether pattern sizes that match WFT spatial resolution capabilities for a given distance capture more WFT than mismatched traps under laboratory trials in which 3 simultaneously presented traps were visible to WFT. We then conducted effectively no-choice trials in commercial polytunnels, in which multiple traps were not visible simultaneously to WFT. For laboratory trials, we predicted that traps designed to be resolvable at a given viewing distance would outperform traps that are either smaller or larger than this predicted value for the given distance. For polytunnel trials, we predicted that increasing pattern size, and therefore the visual range it can be detected over for WFT, would increase trap catch. Together, the results of this study provide a simple method to improve trap design by considering spatial resolution capacity for WFT monitoring.

## Materials and Methods

### WFT Culture

WFT used for laboratory testing were collected from a colony at Swansea University, Swansea, UK. The colony was maintained in a temperature-controlled room at 25 to 27 °C in a chamber made with transparent Perspex siding (30 cm L × 30 cm W × 45 cm H). Chambers contained potted chrysanthemums (*Chrysanthemum* spp.) with runner beans (*Phaseolus coccineus*) provided as egg-laying substrate. Water was added to absorbent matting at the bottom of the rearing chamber to maintain humidity above 60%. To facilitate collection of adult WFT, runner beans containing nymphs and eggs were transferred to smaller plastic containers (15 cm L × 23 cm W × 13 cm H) containing pine pollen, cut chrysanthemum flowers, fresh runner beans, and dampened filter paper to maintain humidity. Boxes were checked every 2 d, allowing for collection of adults of roughly the same age for experimental testing.

### Stimuli Size and Viewing Distance in Laboratory Conditions

Using Equation (1), we predicted the range of resolvable stimuli sizes that could be viewed from a 5, 10, and 20 cm viewing distance for WFT (Table 1). We then designed sticky-trap cards that consisted of a geometric yellow flower shape placed against a blue background. We selected a flower pattern as this shape has been found to be attractive to WFT in a previous study (Ren et al. 2020). We selected flower size based on a predicted IO angle of 14° to help ensure that stimuli would be resolvable to the WFT visual system at the tested distances, while ensuring that selected stimuli sizes were below the resolution threshold for the other viewing distances selected (Table 1).

**Table 1.** Predicted resolvable size of a geometric flower shape for viewing distances used in laboratory trials and an IO angle of 10° to 14° for WFT. We selected the stimuli size for laboratory experiments based on an IO angle of 14°

Distance (cm)	Resolvable sizes (cm)	Stimuli size (cm)
5	0.87 to 1.23	1.23
10	1.75 to 2.46	2.46
20	3.50 to 4.91	4.91

**Fig. 1.**
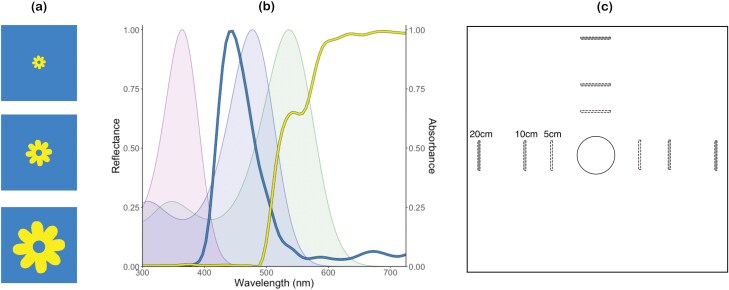
Overview of experimental design for laboratory experiments. A) Small, medium, and large stimuli predicted to be resolvable from a 5, 10, and 20 cm viewing distance, respectively, for the WFT visual system. B) Normalized reflectance spectra of the blue and yellow colors used in sticky traps. Spectral sensitivity curves of WFT are shown with peak sensitivity at 363 nm (purple shaded area), 476 nm (blue shaded area), and 535 nm (green shaded area). C) Overhead view depicting the experimental set up. The central circle represents the release point for WFT at the beginning of experiments, the dashed rectangles represent possible trap position during trials. One trap of each size (small, medium, large) was placed either 5, 10, or 20 cm from the release point, with all traps within a single trial being placed at the same viewing distance.

Sticky cards were designed in Adobe Photoshop (Adobe Inc., USA), using a blue and yellow color previously found to be attractive to WFT (Dearden et al. 2023). Each sticky trap consisted of a 7 × 7 cm blue square with a yellow flower in the center (Fig. 1A). The flower was resized using the free transform tool to be resolvable from either 5, 10, or 20 cm (Table 1), which we will hereafter refer to as small, medium, and large stimuli, respectively. Reflectance spectra of the blue and yellow used for sticky traps were measured using an AvaSpec-ULS2048CL-EVO-UA-25 spectrophotometer (200 to 1,100 nm) with a AvaLight-DH-S Deuterium–Halogen light source (190 to 2,500 nm) using Avasoft software (Avantes, Netherlands). Three measurements were taken from each color and averaged to generate normalized reflectance curves (Fig. 1B). WFT spectral sensitivity curves with lambda max values of 363, 476, and 535 nm (Otani et al. 2014) following a Govardovskii template (Govardovskii et al. 2000) are overlayed with sticky card reflectance template (Fig. 1B). Traps were printed on Koala Photo Satin 190 GSM paper using a Canon Pro 4100S printer, cut to size, and a sticky glue (Horsefly Glue, Sticky-trap Ltd., UK) was applied.

Choice trials to assess the role of viewing distance and stimuli size on trap catch took place in a climate-controlled greenhouse maintained at 24 °C located at Swansea University, Swansea, UK (51.608012, −3.981281) in October 2023. We consider laboratory trials to be choice trials because all 3 presented traps (but not necessarily trap patterns) are predicted to be simultaneously resolvable at all tested distances (ie resolvable stimuli sizes are smaller than trap size). Approximately 50 adult thrips from the lab culture were collected and released into the center of a mesh insect cage (47.5 cm L × 47.5 cm W × 47.5 cm H). One trap of each pattern size (small, medium, and large) was placed at 5, 10, or 20 cm from the release position (Fig. 1C). All traps were placed at the same distance within each trial, so that predicted resolvable distance of one trap pattern would match the initial viewing distance, while there would be a mismatch between the predicted resolvable distance of the other 2 traps with the initial release distance. The position of the traps was randomized between trials to reduce any potential positional effects on subsequent analyses. Traps were positioned flush with the bottom of the mesh cage and held in place using a metal clip embedded in a wooden block, which was placed beside the trap. After 2 h, we collected the sticky traps and counted the number of WFT captured. Three trials, one for each tested distance, were conducted simultaneously. Cages were shaken out and wiped with damp paper towels after each trial to ensure no thrips from previous trials remained. We repeated experiments at each distance 15 times each, for a total *N* = 45.

### Range of Attraction to Trap Pattern in Polytunnels

We performed a similar experiment as presented above in commercial polytunnels growing peppers (*Capsicum annuum*), allowing us to test the effect of stimuli size and viewing distance in a more complex testing environment. Polytunnel trials took place in Antalya, Türkiye (37.041110, 30.853910) between 26 and 28 September 2023. Stimuli were designed to be resolvable by the WFT visual system at 15, 30, and 50 cm viewing distances (hereafter referred to as small, medium, and large traps, respectively) using Equation (1) and an IO angle of 14° (Table 2). The same yellow geometric flower was added to blue background cards as in laboratory experiments; however, sticky traps were increased to 24.5 cm L × 12.5 cm W for the polytunnel experiments. To ensure that differences in trap catch were due to differences in stimuli size, rather than differences in the amount of blue relative to yellow color on a trap, we varied the number of stimuli between small, medium, and large traps for polytunnel experiments (Table 2). We ensured that, for traps with more than 1 flower pattern element, the distance between stimuli pattern elements was larger than the size of each individual pattern element, so that each flower was independently resolvable at the target viewing distance.

**Table 2.** Predicted resolvable size of a geometric flower shape for viewing distances used in polytunnel trials and an IO angle of 10° to 14° for WFT. We selected the stimuli size for polytunnel experiments based on an IO angle of 14°. Sticky traps used for polytunnel trials also differed in the number of flower stimuli per trap in order to maintain a similar ratio of blue:yellow across stimuli sizes. This is expressed as the percentage of each card that is yellow

Distance (cm)	Resolvable sizes (cm)	Stimuli size (cm)	Number stimuli	Percent yellow (%)
15	2.62 to 3.68	3.68	6	20.28
30	5.25 to 7.37	7.37	2	27.07
50	8.75 to 12.28	12.28	1	28.37

**Fig. 2.**
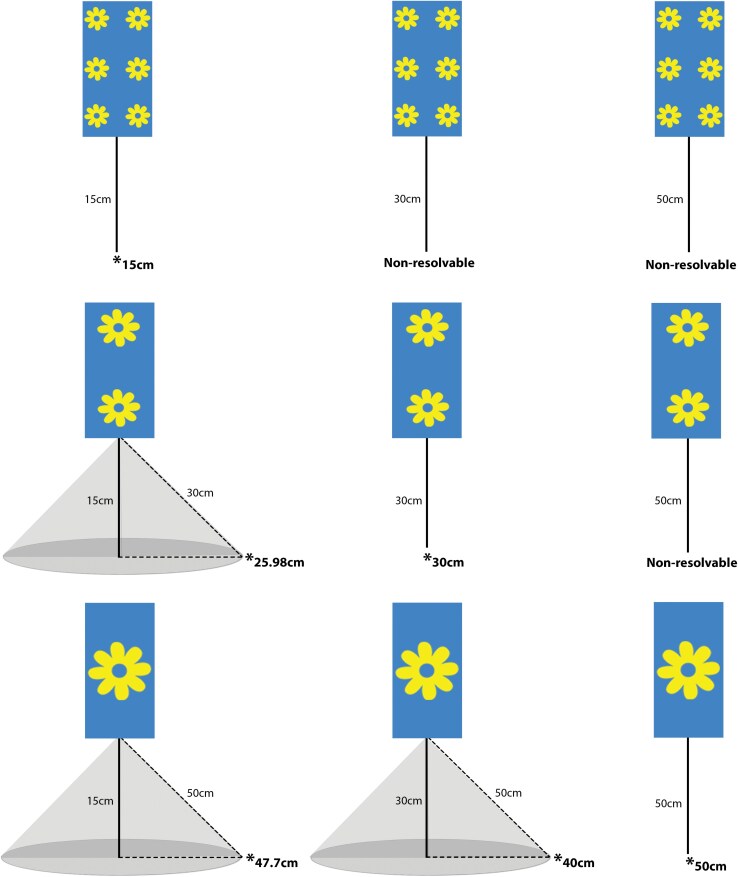
Small, medium, and large flower stimuli for polytunnel testing predicted to be resolvable from a maximum distance of 15, 30, and 50 cm viewing distances, respectively, for the WFT visual system. Whether the flower pattern is predicted to be resolvable is based on stimuli size and the height each trap was positioned above the crop canopy. Given that WFT could view traps from positions either directly below or adjacent to the trap position, we show the range of resolvable distances for each trap type and height. An asterisk indicates the maximum distance for which trap pattern is predicted to be resolvable for WFT and a shaded gray cone represents traps that have resolvable patterns from positions adjacent to the trap location.

Traps were coated in a sticky insect glue (Glue n’Trap, Horse Master, Pommier Nutrition, FR) and hung at either 15, 30, or 50 cm above the crop canopy. In contrast to laboratory experiments where sticky traps were placed in a straight line of site from a fixed release point, sticky traps in polytunnel trials were visible to WFT in a more complex, 3D environment. Given the resolvable distance of each pattern and trap height for small, medium, and large traps, we calculated the maximum distance a trap pattern is resolvable for WFT that are in plants not directly below the sticky trap location (Fig. 2). For small flower patterns, pattern is predicted to be resolvable only for WFT viewing the trap from directly below trap position at the 15-cm trap height. For medium flower patterns, flower pattern is resolvable for WFT located directly below the trap hung at 15 and 30 cm, and from a range of 25.98 cm from the trap location for traps hung at the 15 cm height. For the large flower pattern, pattern is resolvable from directly below the traps at all 3 heights, and from 47.70 and 40.00 cm distances from the trap location for the 15 and 30 cm heights, respectively.

We placed 9 sticky traps, one of each stimulus size and height combination, in a single row of crops with 2.5 m between each trap. We consider polytunnel trials to be effectively no-choice trials because at 2.5 m, calculations of WFT visual resolution indicate the minimum resolvable stimulus size (43.74 to 61.39 cm) is larger than the maximum dimension (27.5 cm) of the trap size used for this experiment, meaning that no more than one trap is predicted to be resolvable at a time. To help reduce potential affects from nonhomogenous distributions of WFT within crops, we replicated this in 4 rows of crops per polytunnel across three polytunnels, for a total *N* = 108, with *n* = 12 for each size and height. Each polytunnel differed slightly in overall size and dimensions, but all 3 consisted of a minimum of 10 rows of crops spaced 2.5 m apart and approximately 30 m long. Two polytunnels were approximately 40 m × 80 m with areas of 0.30 and 0.35 ha and the third polytunnel was approximately 50 m × 80 m with an area of 0.40 ha. Pepper plants were primarily green canopy (not flowering) at the time of experiments, with estimated canopy heights of 65 to 73 cm from the ground. The position of each card within each row was randomized to reduce any positional effects on WFT catch. Traps were taken down after 48 h and the number of WFT on each trap was counted.

### Statistical Analyses

All data analysis was performed with R (ver. 4.3.2.) (R Core Team 2023). For laboratory experiments, we used a Poisson General Linear Model (GLM) to test the effect of stimuli size (factor with 3 levels: small, medium, large) and viewing distance (factor with 3 levels: 5, 10, 20 cm), and their interaction on the number of WFT caught on traps. Model verification indicated underdispersion, so standard errors were corrected using a quasi-GLM model where the variance is given by *φ* × *μ*, where *φ* the dispersion parameter and *μ* is the mean (Zuur et al. 2009). Backwards model selection resulted in a final model with stimuli size, viewing distance, and their interaction as explanatory terms.

Initial data exploration for polytunnel experiments indicated the presence of a single extreme outlier with high influence on the model fit in the dataset (Faraway 2016). We therefore present data analysis methods and results with the outlier point removed in the main text (see Supplemental Materials for model selection with the outlier included). We tested the effect of stimuli size (factor with 3 levels: small, medium, large), height above the canopy (factor with 3 levels: 15, 30, and 50 cm), and their interaction on number of WFT caught. We used the packages glmmTMB (Brooks et al. 2023) to model zero-inflated data structure and DHARMa (Hartig and Lohse 2022) to assess model fit. We assessed whether the inclusion of crop row and polytunnel ID as random effects improved model fit by comparing model AICs. Following selection of the random effect structure, we assessed the significance of single terms and interactions using a backward model selection approach (Zuur et al. 2009). The final model for polytunnel trials included height as the sole fixed effect, polytunnel as a random effect, and a linear negative binomial error structure.

It is also possible that, given the 3D viewing environment possible in polytunnel trials, trap patterns that were visible from positions in the crop canopy adjacent to the trap location might have increased WFT capture, given their wider visible range relative to trap patterns that are only resolvable within a direct line-of-sight (Fig. 2). We, therefore, tested whether WFT catch increased when trap patterns were resolvable from further away (ie increased the potential attractive range of traps). The maximum distance for which trap pattern is predicted to be resolvable was included as a fixed effect, with patterns predicted to be unresolvable for the given viewing distance assigned a value of 0 cm. Data exploration indicated a nonlinear relationship between the maximum resolvable distance and WFT catch, so maximum visibility and its quadratic component were both included as possible fixed effects in the model. Selection of random effect terms indicated the inclusion of crop row nested within polytunnel improved model fit and backwards model selection resulted in a final fixed effect structure with both maximum visible distance and its squared component as predictors of WFT catch.

## Results

### Stimuli Size and Viewing Distance in Laboratory Conditions

**Fig. 3.**
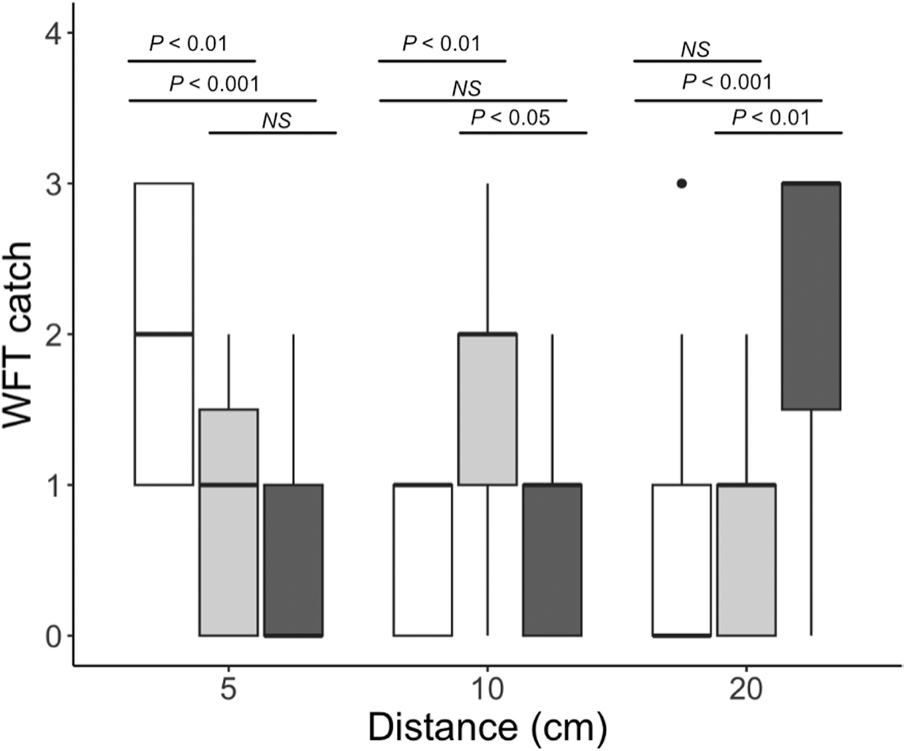
WFT caught on sticky traps with small (white), medium (light gray), and large (dark gray) stimuli designed to be resolvable from 5, 10, and 20 cm viewing distances, respectively, in laboratory experiments. There is a significant interaction between viewing distance and stimuli size on WFT catch (𝜒^2^ = 58.67, df = 4, *P* < 0.001). Posthoc tests indicate that the stimulus size predicted to match the given viewing distance caught significantly more WFT compared to traps with mismatched stimuli sizes for that distance. *P*-values are shown for significant differences following Tukey correction for multiple comparisons. “NS” indicates no significant difference between groups.

Model results showed a significant interaction between stimuli size and trap distance on WFT catch in laboratory trials (Anova: 𝜒^2^ = 58.67, df = 4, *P* < 0.001). We performed posthoc tests with a Tukey correction to compare traps with small, medium, and large stimuli sizes at each viewing distance. At 5 cm, traps with small stimuli caught significantly more than traps with medium (small – medium: *Z* = 3.12, *P* = 0.005) and large stimuli (small – large: *Z* = 4.03, *P* < 0.001), but no significant difference was detected between medium and large stimuli (medium – large: *Z* = 1.32, *P* = 0.39). At 5 cm, mean ± standard error (SE) WFT caught was 2.0 ± 0.2 on traps with small patterns, 0.8 ± 0.2 on medium pattern traps, and 0.5 ± 0.2 on large pattern traps. At 10 cm, traps with the medium stimulus caught significantly more than small (medium – small: *Z* = 3.26, *P* = 0.003) and large stimuli (medium – large: *Z* = 2.43, *P* = 0.04), but no significant difference was detected between small and large sized stimuli (small – large: *Z* = −1.03, *P* = 0.56). Mean ± SE of WFT caught at 10 cm was 0.5 ± 0.1 on traps with small patterns, 1.7 ± 0.3 on medium pattern traps, and 0.8 ± 0.2 on large pattern traps. At 20 cm, traps with large caught significantly more than traps with small (large – small: *Z* = 3.73, *P* < 0.001) and medium stimuli (large – medium: *Z* = 3.55, *P* = 0.001), but no significant difference was detected between small and medium stimuli (small – medium: *Z* = −0.25, *P* = 0.97) (Fig. 3). Mean ± SE of WFT caught at 20 cm was 0.7 ± 0.2 for traps with small patterns, 0.7 ± 0.2 for traps with medium patterns, and 2.1 ± 0.3 for traps with large patterns.

### Range of Attraction to Trap Pattern in Polytunnels

**Fig. 4.**
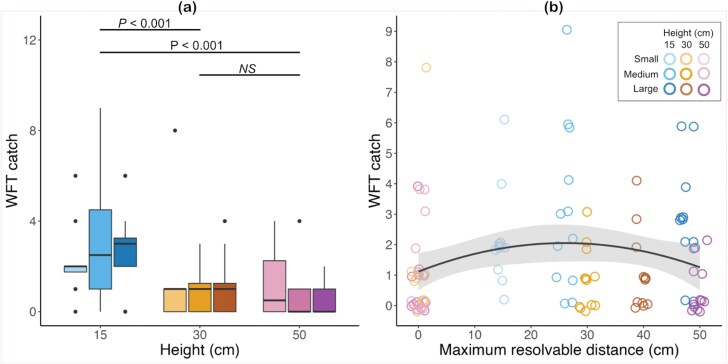
WFT caught on sticky traps in polytunnel experiments. A) Trap height above the crop canopy has a significant effect of WFT catch (𝜒^2^ = 29.92, df = 2, *P* < 0.001), with significantly more WFT caught on traps hung at 15 cm above the crop canopy relative to 30 and 50 cm heights. *P*-values are shown for significant differences following Tukey correction for multiple comparisons. “NS” indicates no significant difference between groups. Note that statistical comparisons are only between heights tested. Traps hung at 15 cm are displayed in blues, at 30 cm in oranges, and at 50 cm in purples. For each color, the small pattern is displayed in the lightest hues, medium pattern in the mid-tone hue, and large patterns in the darkest hue of the respective color (see key in panel (B)). B) The maximum resolvable distance of trap pattern from the crop canopy shows a significant quadratic relationship with WFT catch (𝜒^2^ = 5.68, df = 1, *P* = 0.02), with less WFT caught on traps whose patterns are predicted to be unresolvable (0 cm) and at the largest resolvable distances (50 cm) relative to intermediate values, with a peak at approximately 26 cm. Traps hung at 15 cm are represented in blue hues, at 30 cm in orange hues, and at 50 cm in purple hues. For each color, the small pattern is displayed in the lightest hues, medium pattern in the mid-tone hue, and large patterns in the darkest hue of the respective color. Shaded gray area represents 95% confidence interval for line of best fit.

Backwards model selection indicated no significant interaction between trap size and height (𝜒^2^ = 3.16, df = 4, *P* = 0.53) nor a significant additive effect of size (𝜒^2^ = 0.004, df = 2, *P* = 0.998) on WFT catch, resulting in a final model with trap height as the only fixed effect component for polytunnel trials. There was a significant effect of trap height on WFT capture (Anova: 𝜒^2^ = 29.92, df = 2, *P* < 0.001). Posthoc tests with Tukey correction indicated traps hung at 15 cm caught significantly more thrips that traps hung at 30 cm (15 to 30 cm: *Z* = 3.89, *P* < 0.001) and 50 cm (15 to 50 cm: *Z* = 4.72, *P* < 0.001). There was no significant difference between number of WFT caught on traps hung at 30 and 50 cm heights (30 to 50 cm: *Z* = 1.13, *P* = 0.50) (Fig. 4A). Mean ± SE of WFT caught at 15 cm distances was 2.2 ± 0.4 for traps with small patterns, 3.1 ± 0.8 for traps with medium patterns, and 2.8 ± 0.6 for traps with large patterns. At 30 cm, mean ± SE WFT caught was 1.3 ± 0.7 for small patterns, 0.9 ± 0.3 for medium patterns, and 1.1 ± 0.6 for large patterns. At 50 cm, mean ± SE WFT caught was 1.3 ± 0.5 for small patterns, 0.7 ± 0.3 for medium patterns, and 0.5 ± 0.6 for large patterns. Results with the outlier point included were similar, but with no significant difference between traps hung at 15 and 30 cm (15 to 30 cm: *Z* = 1.67, *P* = 0.22; see Supplemental Materials).

Results of maximum resolvable distance on WFT capture showed a significant effect of resolvable distance (𝜒^2^ = 6.39, df = 1, *P* = 0.01) and its quadratic component (𝜒^2^ = 5.68, df = 1, *P* = 0.02) on WFT catch. Results indicated that WFT caught per trap increase with an increasing resolvable range, up to approximately 26 cm, at which point WFT catch begins to decline up to 50 cm resolvable distances (Fig. 4B).

## Discussion

Our results showed that matching pattern size to the predicted spatial resolution capabilities of WFT significantly improved trap performance relative to mismatched traps in laboratory trials. We found a significant interaction between pattern size and viewing distance in laboratory trials, with pattern sizes designed for a specific viewing distance outperforming both patterns that were smaller than the spatial resolution capacity (ie nonresolvable) and larger than the minimal resolvable pattern size for a given viewing distance (Fig. 3). Paired with polytunnel experiments, which tested attraction over larger distances and in a more complex viewing environment than laboratory tests, we showed that increasing the resolvable distance of patterns increased WFT catch up to approximately 26 cm (Fig. 4B). Together, our results suggest that sticky traps with patterns designed to be resolvable at distances of between 15 and 25 cm can improve monitoring and control of WFT.

Results from the laboratory experiment also provide strong behavioral validation for the spatial resolution abilities of WFT, with pattern sizes that matched the given viewing distance based on predicted interommatidial angles of WFT outperforming traps with a mismatch between pattern size and predicted resolvable distance (Fig. 3). Because the stimuli that captured the most WFT varied with distance, these results cannot be attributed to differences in general attractiveness of each stimulus size. When the yellow pattern on traps was smaller than the predicted resolvable size for the WFT visual system, trap pattern should not be visible; however, the visual system of WFT will still receive input from both the blue background and yellow flower of traps. Previous studies using light-emitting diodes (LEDs) showed that combining green (which stimulates the same photoreceptor cells as yellow light for the WFT visual system) and blue LEDs reduced attraction of WFT relative to either solid blue or green LEDs (Stukenberg et al. 2020). While trap patterns can positively impact trap capture (Mainali and Lim 2010, Dearden et al. 2023), this suggests that patterns that are too small for a target distance may negatively impact trap performance if spatial resolution is not taken into account.

Larger flowers and larger plant densities tend to be more attractive to pollinating insects (Conner and Rush 1996, Dafni et al. 1997, Danderson and Molano-Flores 2010), either because larger flowers function as extreme (supernormal) versions of attractive stimuli (Staddon 1975) or because of correlations between flower size and food rewards (Dafni et al. 1997). Given their role in at least some pollination (Eliyahu et al. 2015), it was also possible that patterns larger than the minimum resolvable size for a given viewing distance could have increased attractiveness to WFT. Though our results showed that these traps were also less attractive than those whose pattern sizes matched the given viewing distance in laboratory trials (Fig. 3). This may explained by the secondary attractiveness of yellow relative to blue colors for WFT (Brødsgaard 1989, Broughton and Harrison 2012, Cruz‐Esteban et al. 2020), with the yellow flower in larger than the minimal resolvable size occupying a larger area of the visual field. In some cases, there are also diminishing responses with increased stimuli size in other cases. For example, experiments using artificial flowers in the hawkmoth *Macroglossum stellatarum* (Lepidoptera: Sphingidae) found that intermediate flower sizes received more visits than the smallest and largest stimuli (Bell 1985) and experiments with the honeybee, *Apis mellifera* (Hymenoptera: Apidae), show that intermediate-sized flower clusters had the highest visitation rates (Akter et al. 2017). These studies clearly demonstrate that there is no universal pattern for attraction to larger stimuli, highlight the benefit of a systematic evaluation of stimuli size in insect attraction. Indeed, spatial acuity may have a role in these results too.

The addition of patterns to colored sticky traps has been shown previously to improve WFT capture relative to solid-colored traps (Mainali and Lim 2010, Dearden et al. 2023). In the current study we used the resolving capabilities of the WFT visual system to systematically choose pattern size for specific testing distances, similar to how prior work has used receptor-based color modeling to identify effective trap colors (Dearden et al. 2023). Spatial resolution capabilities depend on the distance from which stimuli are viewed (Land 1997, Caves et al. 2018). Previous studies have shown that solid color traps presented at distances of 20 to 30 cm outperform traps viewed from further away (Khavanad et al. 2019, Ren et al. 2020). Our results suggested that the addition of a pattern does not increase the attractive range for WFT, with traps placed 15 cm above the crop canopy catching significantly more WFT than traps at 30 and 50 cm above the crop canopy, irrespective of pattern size (Fig. 4A) and predicted resolvable ranges suggesting patterns are most effective up to distances of approximately 26 cm (Fig. 4B). However, the addition of olfactory cues can increase the range of attraction for WFT up to 100 cm, with no significant difference between traps placed at 50 and 30 cm distances when paired with the floral volatile *p*-anisaldehyde (Ren et al. 2020). Multimodal traps incorporating olfactory stimuli and pattern elements may therefore offer opportunities to further increase pest capture at longer ranges. In the absence of olfactory cues however, results from the polytunnel trials did not show an interaction between pattern size and viewing distance on WFT catch, as observed in laboratory trials. This may be because the tested distances in the polytunnel were larger than the apparent effective range of trap pattern for attracting WFT identified in the current study. Experiments measuring whether matching pattern sizes to spatial resolution capabilities at distances less than 26 cm in polytunnel conditions would be a useful next step for testing pattern size in sticky trap design.

The addition of trap patterns designed for the WFT visual system may also increase specificity of trap capture. Beneficial insects, such as crop pollinators and predators of crop pests, are also captured on blue sticky cards (Clare et al. 2000, Chen et al. 2004, Broughton and Harrison 2012), requiring a balance between maximizing pest capture while minimizing detriment to beneficial insects. Differences in spatial resolution capabilities between WFT and common beneficials may offer a solution for bycatch. For example, interommatidial angles for beneficial pollinators and predatory beetles are generally between 1° and 3° (Land 1997, Feller et al. 2021), much lower than the predicted 10° to 14° for WFT. This means that stimuli designed to be resolvable for the WFT visual system will differ to stimuli designed for the much finer resolving power of beneficial insects. Future research on the role of spatial pattern on beneficial bycatch will be useful to assess the role of trap pattern to improve trap specificity in agricultural settings.

Overall, our laboratory and polytunnel studies offer valuable insights into the visual behavior of WFT and practical insights into pest management strategies. Results from lab studies demonstrate the utility of spatial modeling in trap design, with pattern sizes that matched the predicted viewing distance catching significantly more WFT than those that were mismatched at the tested viewing distance. Results of the polytunnel trials demonstrated the functional ranges that patterned traps are effective over in more complex visual environments. Our result support that trap design informed by the spatial resolution capabilities of WFT can improve the monitoring and control of this economically important crop pest.

## Supplementary Material

ieaf049_suppl_Supplementary_Material

## Data Availability

Raw data and scripts associated with analysis are available on Dryad (doi: https://doi.org/10.5061/dryad.1zcrjdg2x).
