# Supplementary material for: Modeling spatial acuity improves trap capture of western flower thrips, Frankliniella occidentalis (Thysanoptera: Thripidae)
Source: J Insect Sci. 2025 May 13;25(3):5. doi: 10.1093/jisesa/ieaf049 (PMC12070478; doi:10.1093/jisesa/ieaf049)
Supplement: ieaf049_suppl_Supplementary_Material [file ieaf049_suppl_supplementary_material.docx]

SUPPLEMENTAL MATERIALS

For the full dataset for field trails, including the outlier value, we included the possible fixed terms of stimuli size (factor with 3 levels: small, medium, large), height above the canopy (factor with 3 levels: 15 cm, 30 cm, and 50 cm), and their interaction on number of western flower thrips (WFT) caught. Comparison of model AICs indicated that the inclusion of row number nested within polytunnel as a random effect improved model fit. Analysis of model residuals using DHARMa (Hartig and Lohse 2022) suggested that a quadratic negative binomial error structure with a zero-inflation component scaled uniformly for all fixed effect parameters best fit the data structure. Backwards model selection indicated no significant interaction between stimuli size and trap height on WFT capture (𝜒2 = 6.97, df = 4, *p* = 0.138) nor a significant additive effect of stimuli size (𝜒2 = 4.74, df = 2, *p* = 0.094) on WFT capture, resulting in a final model with height above the canopy as the sole fixed predictor of WFT capture.

Analysis of field trials indicate a significant effect of trap height on WFT capture. In the full dataset, including the outlier value, there was a significant effect of trap height above the canopy on WFT capture (Anova: 𝜒2 = 14.14, df = 2, *p* < 0.001). Post-hoc comparisons with a Tukey correction indicate a significant difference in WFT catch between traps hung 15 cm above the crop canopy and traps hung 50 cm above the crop canopy (15 cm – 50 cm: *Z* = 3.76, *p* < 0.001), but no significant differences between traps hung at 15 cm and 30 cm (15 cm – 30 cm: *Z* = 1.67, *p* = 0.22) or 30 cm and 50 cm (30 cm – 50 cm: *Z* = 2.13, *p* = 0.08).

REFERENCES

[Software] Hartig F, Lohse L. 2022. DHARMa: Residual Diagnostics for Hierarchical (Multi-Level / Mixed) Regression Models. [accessed 2024 Jan 14]. https://cran.r-project.org/web/packages/DHARMa/index.html.
